# Supplementary material for: Environmental arginine controls multinuclear giant cell metabolism and formation
Source: Nat Commun. 2020 Jan 22;11:431. doi: 10.1038/s41467-020-14285-1 (PMC6976629; doi:10.1038/s41467-020-14285-1)
Supplement: Supplementary file 1 — Supplementary Information [file 41467_2020_14285_MOESM1_ESM.pdf]

Supplementary Information

Environmental Arginine Controls Multinuclear Giant Cell Metabolism and Formation

Brunner J. S., et al.

Supplementary figures

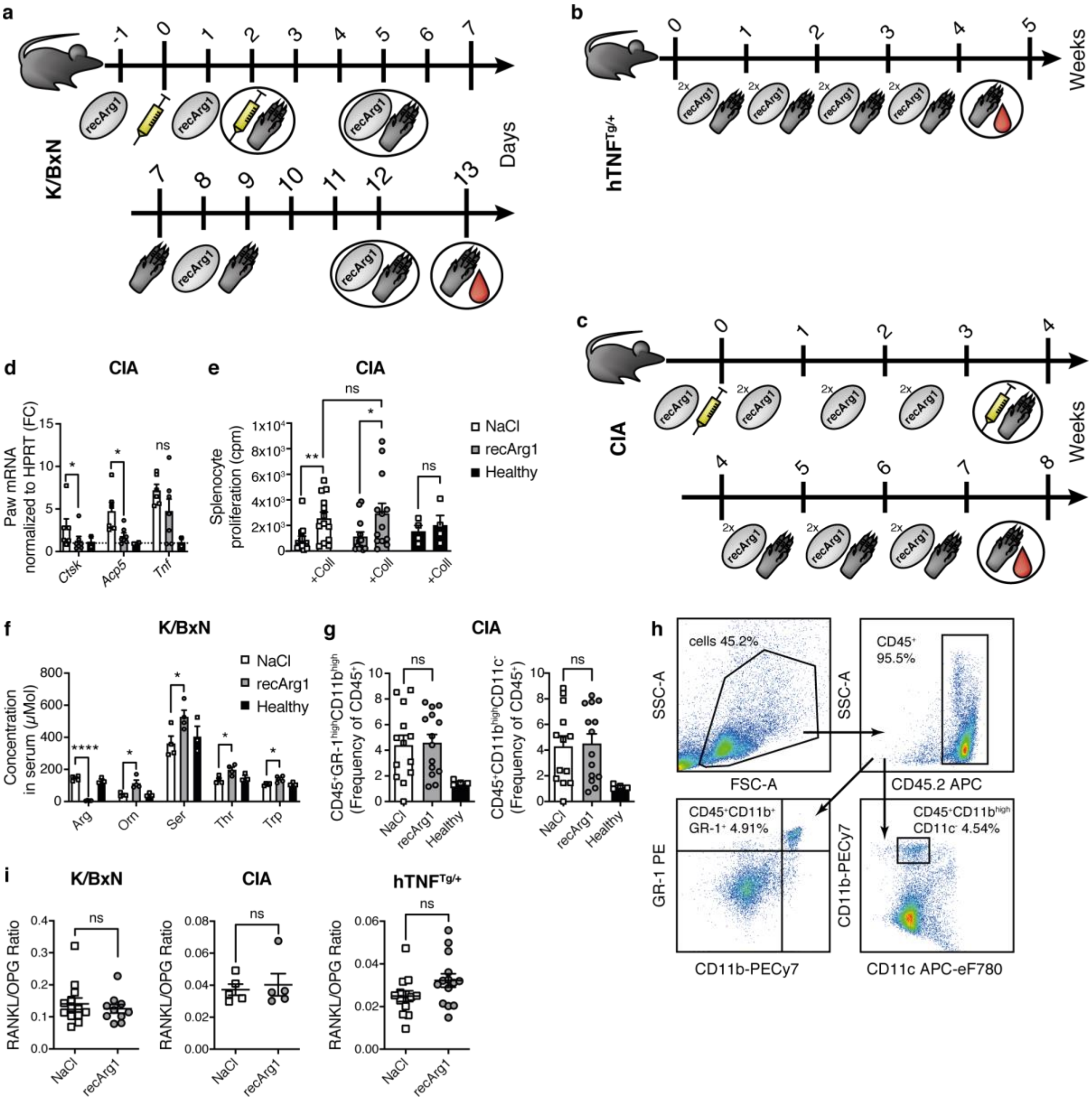

Supplementary Figure 1. Effects of recArg1 on serum amino acids, myeloid cell precursor levels and RANKL/OPG ratios during murine arthritis. **a** Scheme depicting workflow of K/BxN model in Fig. 1a. Animals were injected with 50 mg/kg recArg1 or saline (NaCl) one day prior to K/BxN serum injection. Mice were treated and scored twice weekly and harvested at day 13. **b** Scheme depicting workflow of hTNF$^{Tg/+}$ model in Fig. 1b. Animals were injected at 5 weeks of age, twice weekly with 50 mg/kg recArg1 or NaCl and scored once per week

until harvest, post 5 weeks. **c** Scheme depicting workflow of CIA model in Fig. 1c. Mice were injected with 50 mg/kg recArg1 or NaCl one day prior to CIA induction and were continuously treated twice weekly, scored once per week until harvest, post 8 weeks. **d-e** Osteoclast specific markers in paws (**d**) and splenocyte proliferation post type II collagen (Coll) stimulation (**e**) of mice suffering from CIA in Fig. 1c (**d** healthy *n*=2, NaCl *n*=6, recArg1 *n*=7; **e** healthy *n*=4 NaCl, recArg1 *n*=13). **f** Selected serum amino acids of mice suffering from K/BxN arthritis (healthy *n*=3, NaCl, recArg1 *n*=4). **g** Splenic myeloid populations of mice suffering from collagen induced arthritis (CIA) (healthy *n*=4, NaCl, recArg1 *n*=13). **h** Gating strategy related to g. **i** Serum RANKL/osteoprotegerin (OPG) ratio of arthritic mice (K/BxN: NaCl *n*=13, recArg1 *n*=10; CIA: NaCl, recArg1 *n*=5; hTNF$_{Tg/+}$: NaCl *n*=13, recArg1 *n*=14). Data are mean ± SEM, *P < 0.05, **P < 0.01, ****P < 0.0001, unpaired t-test (e-f, h), one-way ANOVA Tukey post-hoc test (g). Source data are provided as a Source Data file.

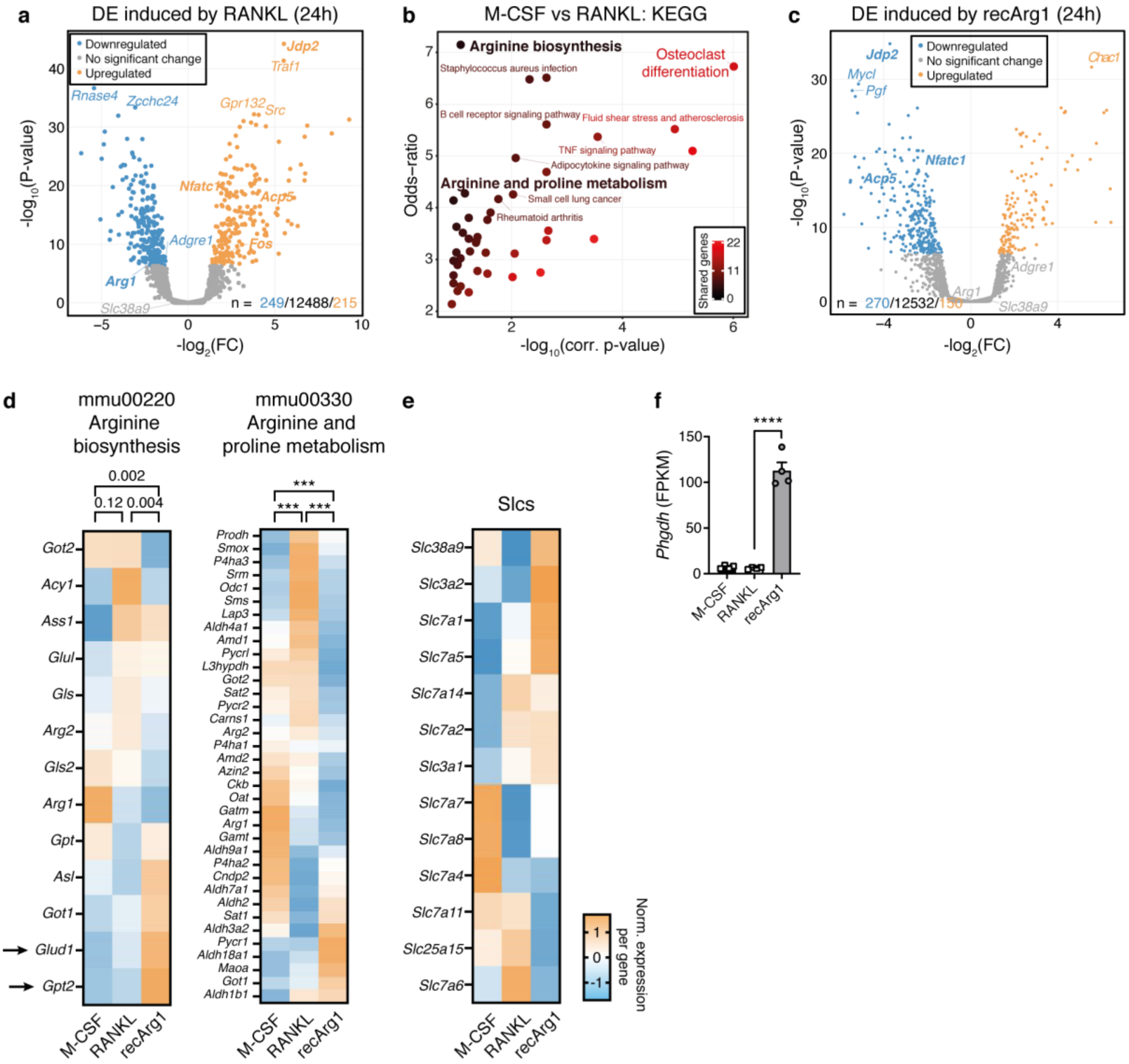

Supplementary Figure 2. Transcriptional profiling reveals the importance of arginine for RANKL signalling. **a** Volcano plot of significantly differentially expressed (DE) genes (log_2FC) plotted against adj. p-value between M-CSF versus RANKL for 24h (*n*=4). **b** KEGG enrichment analysis of **a**. **c** Volcano plot of significantly DE genes (log_2FC) plotted against adj. p-value between M-CSF/RANKL versus M-CSF/RANKL/recArg1 for 24h (*n*=4). **d** Transcriptional profiles (average normalized expression per gene) of selected KEGG pathways changed upon RANKL signalling in the presence and absence of recArg1. Arrows represent pyruvate synthesis genes. Data represent average of *n*=4 per condition, empirical p-values reported. **e** Transcriptional profiles (average normalized expression per gene) of selected

arginine transporters changed upon RANKL signalling in the presence and absence of recArg1. Significance levels correspond to the probability that the expression values in samples are independent of the actual condition, for the given pathway, according to an empirical permutation test. Data represent average of n=4 per condition. **f** Increased mRNA abundance of *Phgdh* during RANKL signalling in the context of Arg-Depletion (n=4). Data are mean ± SEM, ****$P < 0.0001$, one-way ANOVA Tukey post-hoc test (f). Source data are provided as a Source Data file.

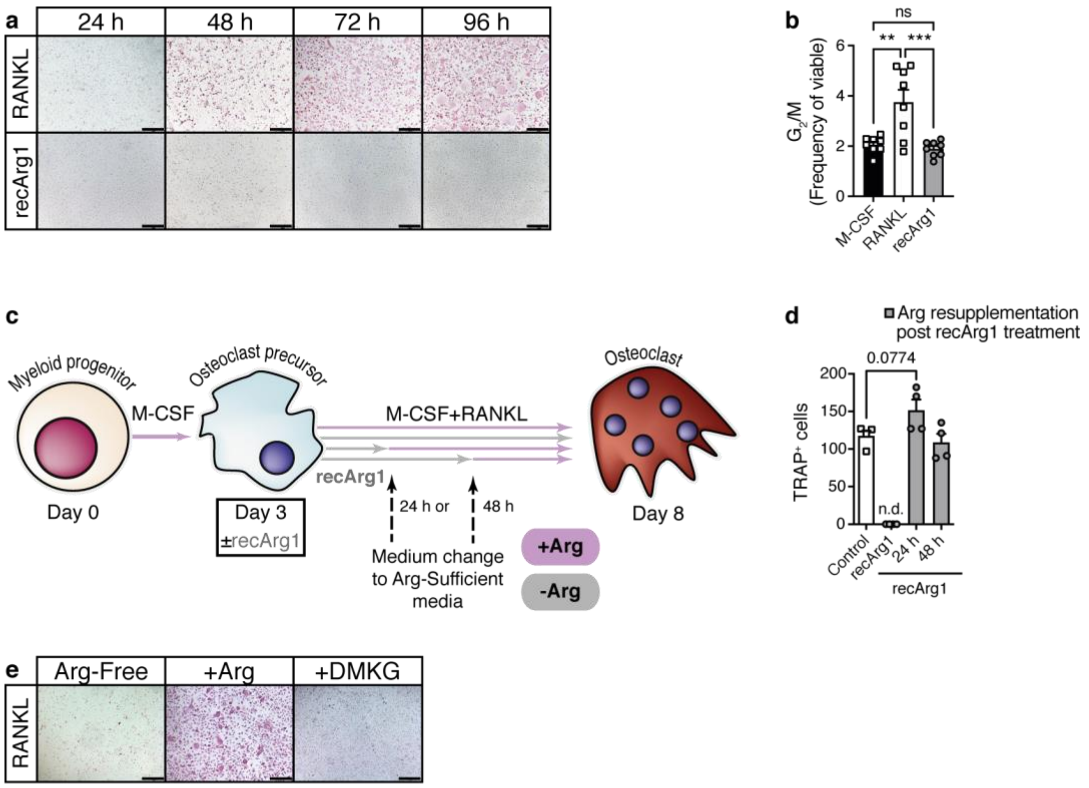

Supplementary Figure 3. Early effects of recArg1 during osteoclastogenesis that are associated with halts in RANKL-dependent G_2/M progression are restored by arginine, but not α-ketoglutarate supplementation. **a** Preosteoclasts were seeded at day 3 of osteoclastogenesis and incubated with M-CSF/RANKL ± 1 µg/ml recArg1. Cells were stained for TRAP-positivity at the respective timepoints, scale bar 200 µm (*n*=4). **b** Quantification of cells in G_2/M phase of the cell cycle 24h post treatment (*n*=8). **c-d** Scheme depicting experimental workflow (**c**) and respective results (*n*=4) (**d**). Pink depicts Arg-sufficient and grey Arg-deficient conditions. **e** TRAP stainings of preosteoclasts in Arg-Free Media supplemented with either arginine (Arg) or dimethyl α-ketoglutarate (DMKG) (*n*=4). Data are mean ± SEM, $**P < 0.01$, $***P < 0.001$, t-test (d), one-way ANOVA Tukey post-hoc test (b). Scale bar represents 200 µm (a, e). Source data are provided as a Source Data file.

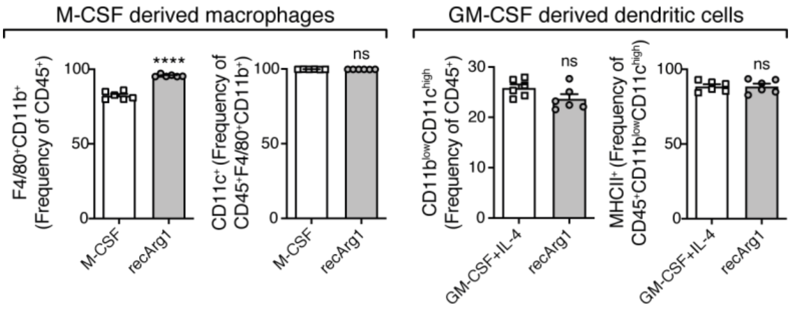

Supplementary Figure 4. RecArg1 does not attenuate macrophage or dendritic cell differentiation. Myeloid precursors were differentiated with M-CSF or granulocyte-macrophage colony-stimulating factor (GM-CSF)/IL-4 into macrophages or dendritic cells respectively in the presence and absence of recArg1 (*n*=6). Data are mean ± SEM. ****P < 0.0001, unpaired t-test. Source data are provided as a Source Data file.

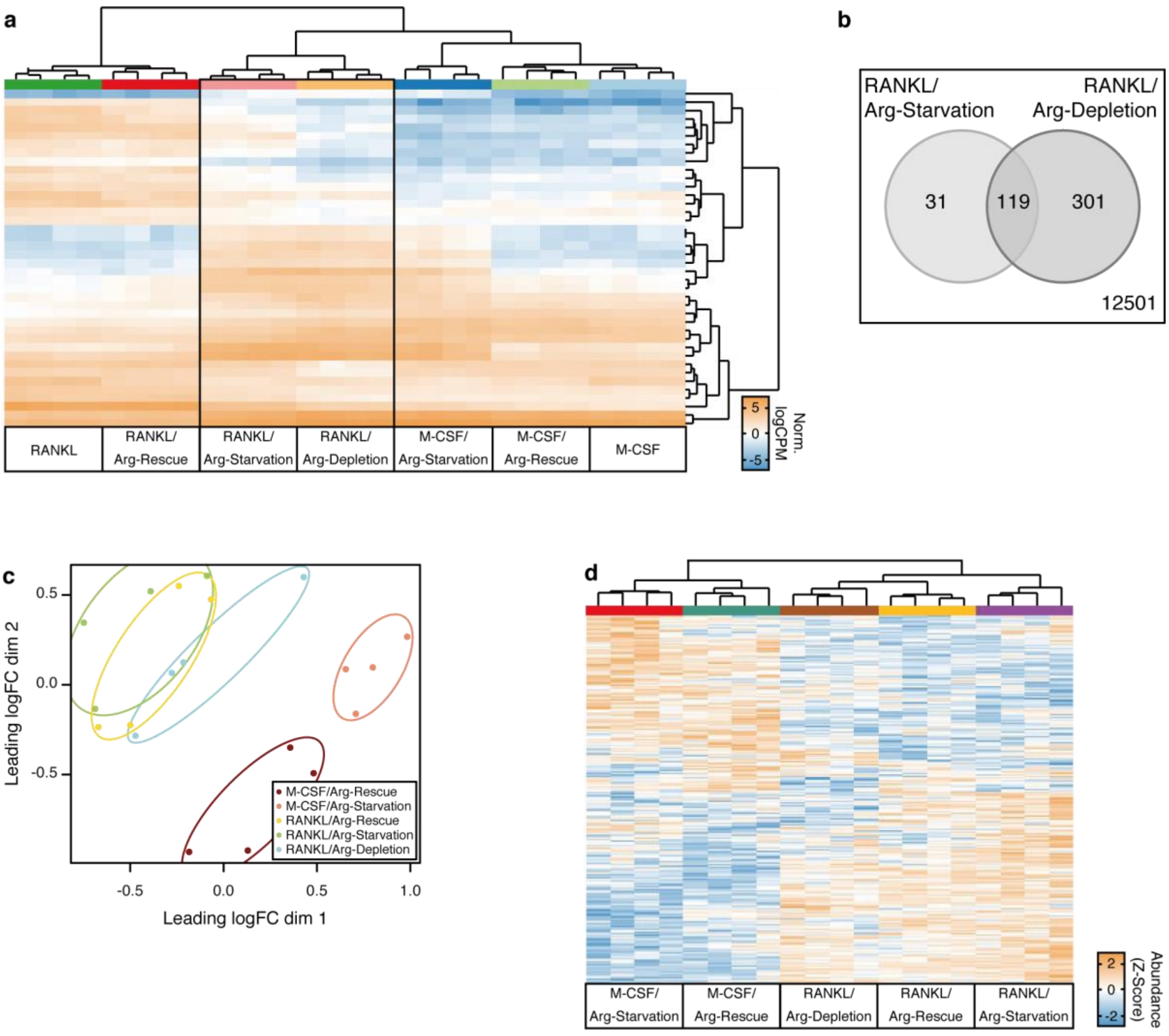

Supplementary Figure 5. Transcriptional and proteomic changes elicited through recArg1 treatment (RANKL/Arg-Depletion) are largely shared with arginine starvation (RANKL/Arg-Starvation). **a** Heatmap of the 40 genes with highest expression difference between RANKL and RANKL/Arg-Depletion in the transcriptomics data in Fig. 3a. Each biological replicate per condition is depicted (*n*=4). **b** Unique and overlapping DE genes in Arg-Starvation and Arg-Depletion conditions (*n*=4). **c** MDS of proteomics data depicted in Fig. 3a. **d** Complete heatmap of proteomics data in Fig. 3d.

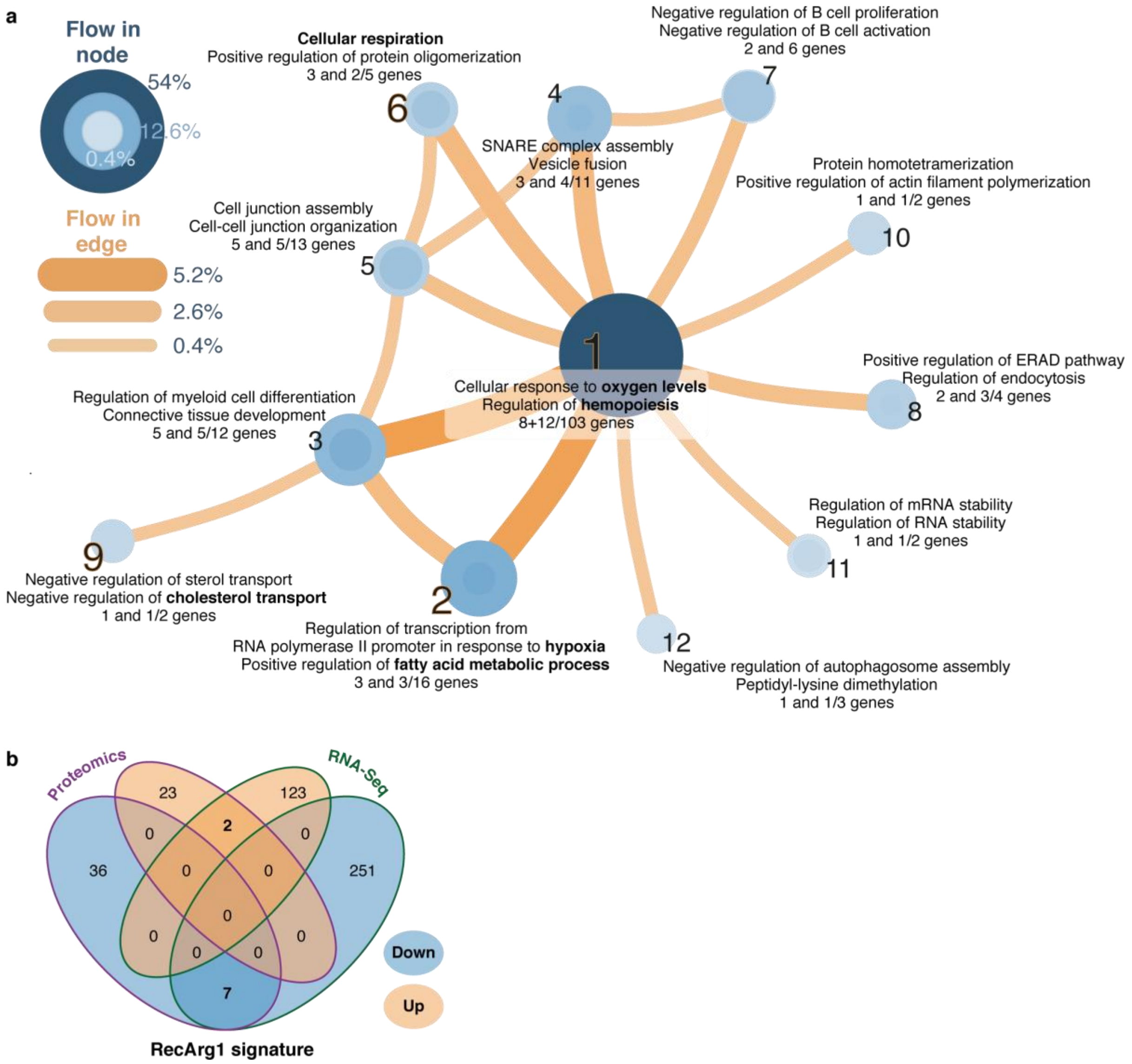

Supplementary Figure 6. Network analysis unifies and accounts for minor overlaps in transcriptomics/proteomics data revealing metabolic hubs are central for arginine dependent RANKL cellular programming. **a** Infomap communities[71] summarizing gene pathways most-likely perturbed by recArg1 or by the arginine dependent effect of RANKL. Edge and node sizes are proportional to the information flow root. Annotations depict the most-enriched GO Biological Processes for the corresponding genes and how many are annotated out of the total gene cluster size. **b** Venn diagram depicting overlaps between the effects of recArg1 on the RANKL proteome and transcriptome.

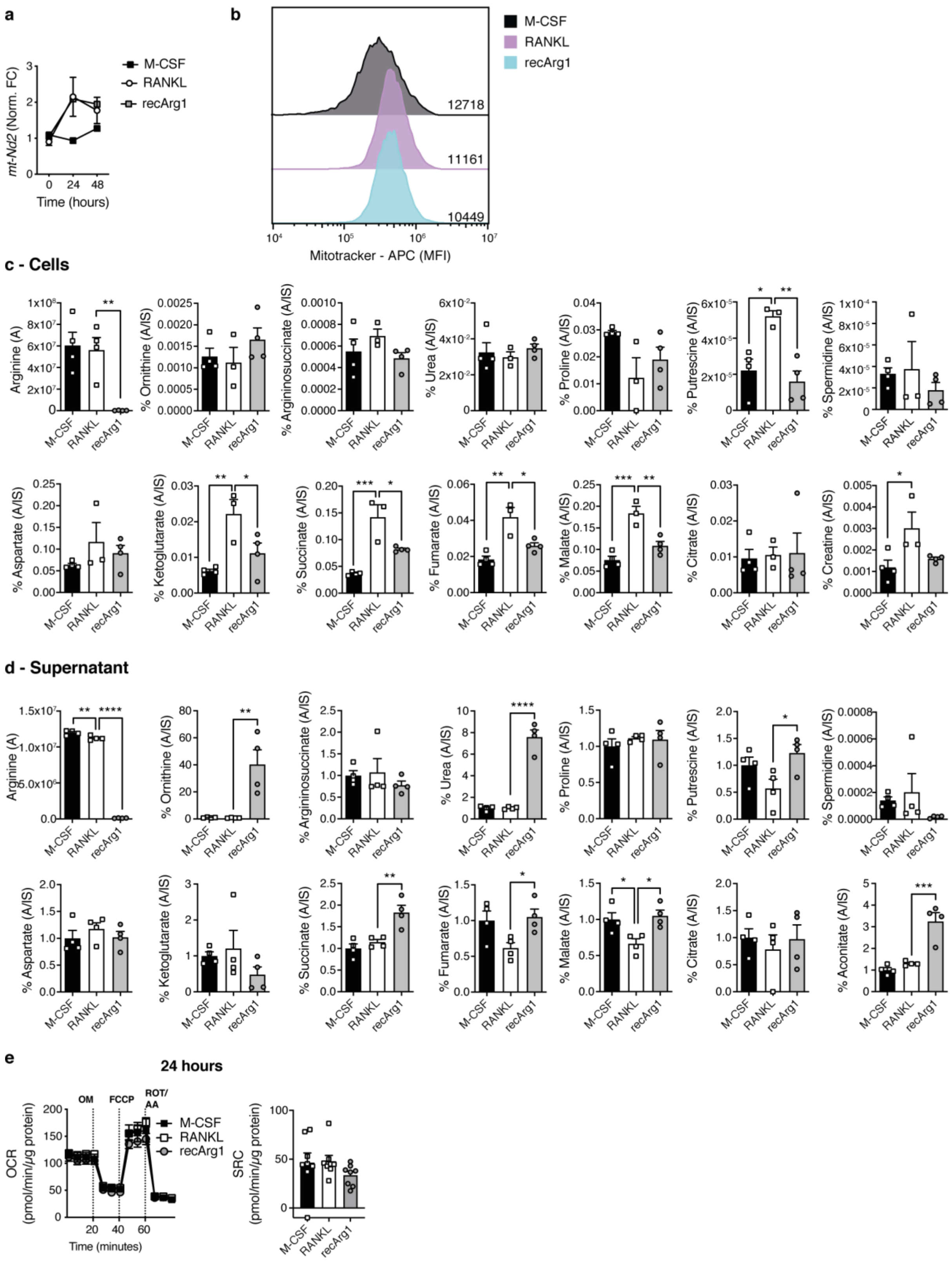

Supplementary Figure 7. Effects of arginine depletion on RANKL-induced mitochondrial biogenesis and metabolite accumulation. **a** qRT-PCR time course of preosteoclast mitochondrial DNA gene *mt-ND2* under the indicated conditions (*n*=4). **b** Preosteoclasts were

treated with the indicated conditions for 48h and stained with MitoTracker Green. Depicted is a representative histogram of F4/80+ cells. **c-d**, Normalized peak area of selected RANKL triggered intracellular (**c**) and extracellular (**d**) metabolite changes related to the TCA and urea cycle and effects of arginine deprivation herein (*n*=4, RANKL intracellular *n*=3). **d** Data are identical to those depicted in Fig. 4f. **e** Oxygen consumption rate (OCR) and spare respiratory capacity (SRC) of preosteoclasts ± recArg1 post 24h (*n*=8). Data are mean ± SEM, *P < 0.05, **P < 0.01, ***P < 0.001, ****P < 0.0001, one-way ANOVA Tukey post-hoc test (c, d). Source data are provided as a Source Data file.

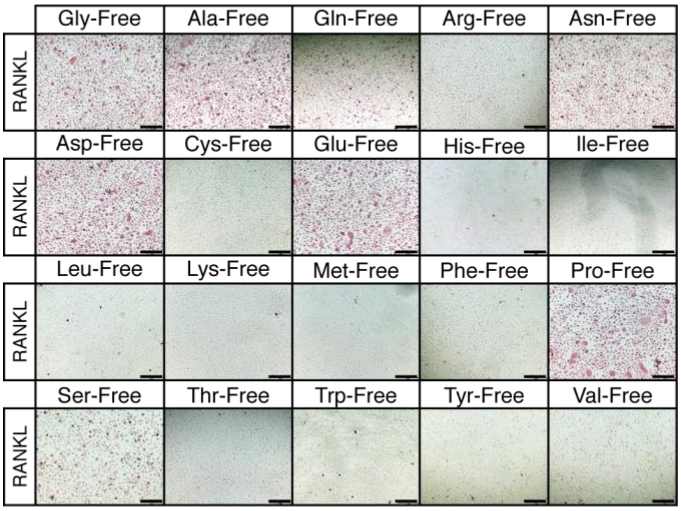

Supplementary Figure 8. Representative TRAP stainings of preosteoclasts cultured in the indicated single AA-Free medium. Data correspond to those in Fig. 6b (*n*=4). Scale bar represents 200 µm.

Supplementary Table 1 Amino acids used for *in vitro* experiments

#	Name	Concentration in MEMα (mg/L)	Concentration in MEMα /Concentration used (mM)	Sigma-Aldrich #
1	Glycine	50.0	0.6667	G5417
2	L-Alanine	25.0	0.2809	A7469
3	L-Alanyl-L-Glutamine	406.0	1.8710	A8185
4	L-Arginine	105.0	0.4976	A8094
5	L-Asparagine	50.0	0.3788	A0884
6	L-Aspartic acid	30.0	0.2256	A7219
7	L-Cysteine hyfrochloride-H2O	100.0	0.5682	778451
8	L-Cystine	24.0	0.1000	C7602
9	L-Glutamic Acid	75.0	0.5102	G5667
10	L-Histidine	31.0	0.2000	H6034
11	L-Isoleucine	52.4	0.4000	I2752
12	L-Leucine	52.4	0.4000	L8912
13	L-Lysine	58.0	0.3973	62840
14	L-Methionine	15.0	0.1007	M9625
15	L-Phenylalanine	32.0	0.1939	P2126
16	L-Proline	40.0	0.3478	P5607
17	L-Serine	25.0	0.2381	S4311
18	L-Threonine	48.0	0.4034	T8441
19	L-Tryptophan	10.0	0.0490	T0254
20	L-Tyrosine	36.0	0.1989	T3754
21	L-Valine	46.0	0.3932	V0500
22	Argininosuccinic acid disodium salt	/	0.500	A5707
23	L-Citrulline	/	0.500	C7629
24	L-Ornithine	/	0.500	O2375
25	Urea	/	0.500	U5378
26	Phenylpyruvate (Phenylpyruvic acid)	/	0.500	286958
27	Alpha-ketoisocaproate (Sodium 4-methyl-2-oxovalerate)	/	0.500	K0629
28	Ketoisoleucine (3-Methyl-2-oxopentanoic acid sodium salt)	/	0.500	198978
29	Spermidine	/	0.010	S0266
30	Putrescine	/	0.500	P5780
31	Dimethyl α-ketoglutarate	/	0.500	349631

Supplementary Table 2 Correspondence between assay and edge weight used for the BioGRID interaction network

Assay	Weight	Assay	Weight	Assay	Weight
Affinity Capture-Luminescence	0.2	Dosage Growth Defect	0	Protein-peptide	0.99
Affinity Capture-MS	0.2	Dosage Rescue	0	Protein-RNA	0.2
Affinity Capture-RNA	0.2	Far Western	0.99	Proximity Label-MS	0.2
Affinity Capture-Western	0.2	FRET	0.99	Reconstituted Complex	0.99
Biochemical Activity	0.99	Negative Genetic	0	Synthetic Growth Defect	0
Co-crystal Structure	0.2	PCA	0.99	Synthetic Haploinsufficiency	0
Co-fractionation	0.2	Phenotypic Enhancement	0	Synthetic Lethality	0
Co-localization	0.2	Phenotypic Suppression	0	Synthetic Rescue	0
Co-purification	0.2	Positive Genetic	0	Two-hybrid	0.99

Supplementary Table 3 Genes contained in clusters related to Figure S6a

#	Name	#	Name	#	Name	#	Name
1	Fancd2	1	Rps11	1	Hsp90b1	1	Ddx47
1	Tcf3	1	Ikbkb	1	Syncrip	1	Usp19
1	Lmo2	1	Drg1	1	Fadd	1	Pspc1
1	Pou5f1	1	Mpo	1	Sec61a1	1	Ppm1h
1	Myod1	1	Hif1a	1	Iqgap1	1	Prrc2c
1	Ube2i	1	Itgb1bp2	1	Clip1	1	Cpne2
1	Nfkbia	1	Zfp106	1	Anxa1	1	Tacc3
1	Ivns1abp	1	Fas	1	Rnf123	1	Adck3
1	Fbxo32	1	Cul3	1	Fubp3	1	Nrp2
1	Zdhhc6	1	Sowahc	1	Lnx1	1	Ehd1
1	Hoxa9	1	Map2k1	1	Farsa	1	Ktn1
1	Psme3	1	Hspa5	1	Msh2	1	Tgfbi
1	Jade1	1	Top2a	1	Thop1	1	Myo1e
1	Fxr1	1	Ywhaq	1	Srp72	1	C1qc
1	Hsp90aa1	1	Grb2	1	Sbds	1	Sqrdl
1	Cited4	1	Hnrnpk	1	Sugt1	1	Hfe
1	Dmrtc2	1	Dhx9	1	Ckap5	1	Serpinb1a
1	Id2	1	Rpl28	1	Psmb5	1	Acsl1
1	Sox15	1	Herc2	1	Atg7	1	Snx2
1	Hoxd13	1	E130012A19Rik	1	Rcc1	1	Cd97
1	Ncoa2	1	Top2b	1	Sall2	1	Emr1
1	Uhrf1	1	Psmc3	1	Ogt	1	Epx
1	Nr3c1	1	Sall4	1	Pdia4	1	Lig1
1	Hyou1	1	Flnb	1	Nup214	1	Pes1
1	Carm1	1	Akap8	1	Fkbp4	1	Asns
1	Smad2	1	Snd1	1	Tecpr1	2	Nfe2

#	Name	#	Name	#	Name
2	Zfp219	3	Zbtb7a	6	Bid
2	Mybbp1a	4	Foxl2	6	Arhgdia
2	Mafg	4	Pias2	6	Bax
2	Ppargc1b	4	Nfkb1	6	Cox4i1
2	Nr1h3	4	Xrcc6	7	Dok1
2	Sufu	4	Sp100	7	Fcgr2b
2	Zfp111	4	Gtf2i	7	Inpp5d
2	Sirt2	4	Uvrag	7	Trim21
2	Med21	4	Nfkb2	7	Pstpip1
2	Rbpj	4	Vps18	7	Lpxn
2	Nfe2l2	4	Vps41	8	Hspa8
2	Srebf1	4	Tgfbrap1	8	Ubqln2
2	Fzr1	5	Cblb	8	Cd47
2	Brd2	5	Dlg4	8	Sgta
2	Rrm2	5	Lck	9	Irak1
3	Atf2	5	Ube2c	9	Pin1
3	Jun	5	Dlg1	10	Lasp1
3	Barx2	5	Rac1	10	Vasp
3	Pbx4	5	Iqgap2	11	Elavl4
3	Creb1	5	Nedd4l	11	G3bp1
3	Brca1	5	Rcc2	12	Sumf1
3	Jdp2	5	Adam10	12	Ehmt2
3	Stat3	5	Fscn1	12	Sumf2
3	Mapk14	5	Pde2a		
3	Sox9	5	Syne1		
3	Bag1	6	Casp3		

Supplementary Table 4 Monitored metabolites of GC-MS analysis

**Metabolite**	**RT (min)**	**m/z**		**Metabolite**	**RT (min)**	**m/z**
Proline	6.9	188		Arginosuccinate	15.8	607
Urea	8.5	205		Agmatine	16.6	330
Serine	8.9	250		Putrescine	16.6	377
Glycerol ($^{13}C_3$) IS	9.5	312		Ornithine	16.7	349
Glycine	10.2	292		Aconitate	16.7	391
Succinate	10.3	263		Citrate	17.3	481
Fumarate	11.2	261		Cadaverine	17.4	391
Alanine	11.4	306		Histamine	17.7	328
Aspartic acid	12.9	278		Tyramine	18.0	354
Malate	14.1	351		Lysine	18.1	435
Creatine	14.8	330		Citrulline	19.3	392
α-Ketoglutarate	15.1	320		Tryptophan	19.9	355
Glutamate	15.6	364		Spermidine	20.2	506
