# Supplementary material for: Environmental arginine controls multinuclear giant cell metabolism and formation
Source: Nat Commun. 2020 Jan 22;11:431. doi: 10.1038/s41467-020-14285-1 (PMC6976629; doi:10.1038/s41467-020-14285-1)
Supplement: Supplementary file 3 — Reporting Summary [file 41467_2020_14285_MOESM3_ESM.pdf]

nature research

Corresponding author(s): Blüml S. & Schabbauer G.

Last updated by author(s): Dec 11, 2019

Reporting Summary

Nature Research wishes to improve the reproducibility of the work that we publish. This form provides structure for consistency and transparency in reporting. For further information on Nature Research policies, see Authors & Referees and the Editorial Policy Checklist .

Statistics

For all statistical analyses, confirm that the following items are present in the figure legend, table legend, main text, or Methods section.

n/a	Confirmed	
☐	☒	The exact sample size (*n*) for each experimental group/condition, given as a discrete number and unit of measurement
☐	☒	A statement on whether measurements were taken from distinct samples or whether the same sample was measured repeatedly
☐	☒	The statistical test(s) used AND whether they are one- or two-sided<br>*Only common tests should be described solely by name; describe more complex techniques in the Methods section.*
☐	☒	A description of all covariates tested
☐	☒	A description of any assumptions or corrections, such as tests of normality and adjustment for multiple comparisons
☐	☒	A full description of the statistical parameters including central tendency (e.g. means) or other basic estimates (e.g. regression coefficient) AND variation (e.g. standard deviation) or associated estimates of uncertainty (e.g. confidence intervals)
☐	☒	For null hypothesis testing, the test statistic (e.g. *F*, *t*, *r*) with confidence intervals, effect sizes, degrees of freedom and *P* value noted<br>*Give P values as exact values whenever suitable.*
☒	☐	For Bayesian analysis, information on the choice of priors and Markov chain Monte Carlo settings
☒	☐	For hierarchical and complex designs, identification of the appropriate level for tests and full reporting of outcomes
☐	☒	Estimates of effect sizes (e.g. Cohen's *d*, Pearson's *r*), indicating how they were calculated

Our web collection on statistics for biologists contains articles on many of the points above.

Software and code

Policy information about availability of computer code

Data collection	Xcalibur software<br>Step-OnePlus Real-Time PCR Software<br>FluorChem HD2<br>Gen5<br>Wave Desktop Software 2.6.1.<br>CytExpert 2.3
Data analysis	Microsoft Excel<br>TraceFinder 4.1 software<br>Cell Profiler 3.1.8<br>FlowJo 10<br>CytExpert 2.3<br>CASAVA<br>MaxQuant software<br>Prism 8<br>Adobe Illustrator CS6<br>Adobe Photoshop CS6<br>Perseus 1.6.1.3<br>R version 3.4.0, 3.5.1<br>ggplot2 3.1.0<br>heatmaply 0.15.2<br>OmicsIntegrator 0.3.1<br>Infomap Python v1

October 2018

Cytoscape 3.6.1
Rsubread 1.26.2
limma 3.36.5
edgeR 3.24.0
clusterProfiler 3.8.1

For manuscripts utilizing custom algorithms or software that are central to the research but not yet described in published literature, software must be made available to editors/reviewers. We strongly encourage code deposition in a community repository (e.g. GitHub). See the Nature Research guidelines for submitting code & software for further information.

Data

Policy information about availability of data

All manuscripts must include a data availability statement. This statement should provide the following information, where applicable:

- Accession codes, unique identifiers, or web links for publicly available datasets
- A list of figures that have associated raw data
- A description of any restrictions on data availability

RNA-Seq data have been deposited in Gene Expression Omnibus (GEO) under accession number GSE125101. The mass spectrometry proteomics data have been deposited to the ProteomeXchange Consortium via the PRIDE65 partner repository with the dataset identifier PXD012405. All code used in the proteomics and transcriptomics differential analyses and subsequent integration is available on GitHub (DOI: 10.5281/zenodo.2541644). Associated figures include Fig. 2b; Fig. 3; Fig. 4g; Supplementary Fig. 2; Supplementary Fig. 5; Supplementary Fig. 6;

Field-specific reporting

Please select the one below that is the best fit for your research. If you are not sure, read the appropriate sections before making your selection.

[x] Life sciences [ ] Behavioural & social sciences [ ] Ecological, evolutionary & environmental sciences

For a reference copy of the document with all sections, see nature.com/documents/nr-reporting-summary-flat.pdf

Life sciences study design

All studies must disclose on these points even when the disclosure is negative.

Sample size	Sample size was chosen to reach statistical significance and was based on other studies with similar methodologies. Sample sizes for animal experiments was calculated and approved by the local ethics committee.
Data exclusions	Statistical outliers have been excluded based on alpha=0.05 on Prism 8 software (GraphPad, La Jolla, CA) for human subjects in Fig. 1; Exclusion criteria for the hTNFTg/+ control group was a combined clinical score lower than 5, at 10 weeks of age.
Replication	All experiments were performed at least two times and represent reproducible findings, except OMICS data and if stated otherwise.
Randomization	Mice were grouped randomly per cage.
Blinding	Arthritis and histological scores were recorded by a blinded, experienced rheumatologist.

Reporting for specific materials, systems and methods

We require information from authors about some types of materials, experimental systems and methods used in many studies. Here, indicate whether each material, system or method listed is relevant to your study. If you are not sure if a list item applies to your research, read the appropriate section before selecting a response.

Materials & experimental systems

n/a	Involved in the study	
[ ]	[x]	Antibodies
[x]	[ ]	Eukaryotic cell lines
[x]	[ ]	Palaeontology
[ ]	[x]	Animals and other organisms
[ ]	[x]	Human research participants
[x]	[ ]	Clinical data

Methods

n/a	Involved in the study	
[x]	[ ]	ChIP-seq
[ ]	[x]	Flow cytometry
[x]	[ ]	MRI-based neuroimaging

Antibodies

Antibodies used	The following antibodies were used for western blotting: p-pS6K T389 (Cell Signaling #9234, 108D2), 4EBP1 (Cell Signaling #9452), p-mTOR Ser2448 (Cell Signaling #5536, D9C2), total S6K (Cell Signaling #2708, 49D7), Grb2 (BD #610112, 81), p-eIF2a XP

Ser51 (Cell Signaling #3398, D9G8), p-4EBP1 Thr37/46 (Cell Signaling #2855, 236B4), Tubulin (Cell Signaling #3873, DM1A), Arginase-1 (Merck #ABS535), anti-igG-horseradish peroxidase-linked (HRP) secondary antibodies (GE Healthcare #NA934, anti-rabbit IgG; #NA931, anti-mouse IgG; anti-chicken IgY #G135A).
The following antibodies were used for FACS/Flow cytometry: CD14-PerCP-Cy5.5 (eBioscience #45-0149, 61D3), F4/80-BV421 (BioLegend #123131, BM8), CD45.2-APC (TONBO #20-0454, 104), CD11c-APC-eF780 (eBioscience #47-0114, N418), CD11b-PeCy7 (eBioscience #25-0112, Clone M1/70), MHC Class II-PE (TONBO #50-5321, M5/114.15.2), F4/80-BV421 (BioLegend #123131, BM8), CD45.2-APC, F4/80-FITC (BioLegend #123108, BM8), CD11b-PeCy7 and GR-1-PE (Ly-6G/Ly-6C, BioLegend #108408, RB6-8C5).

Validation	All antibody are commercially available and their validation statements are available on the manufacturer's website.

Animals and other organisms

Policy information about studies involving animals; ARRIVE guidelines recommended for reporting animal research

Laboratory animals	Female wildtype animals (C57BL/6J, RRID: IMSR_JAX:000664 and DBA/1J, RRID: IMSR_JAX:000670) used for in vivo arthritis models were purchased from Charles River Laboratories and housed in specific-pathogen-free animal facilities of the Medical University Vienna. Mice were 8-9 weeks old at the start of the experiment. hTNFTg/+ mice were a gift from George Kollias. Treatment in these mice started at 5-6 weeks of age.
Wild animals	N/A
Field-collected samples	N/A
Ethics oversight	All animal procedures were approved by the local ethics committee of the Medical University Vienna (BMWFW-66.009/0013-V/3b/2019 and BMWFW-66.009/0227-WF/V/3b/2017) and were conducted in strict accordance with Austrian law.

Note that full information on the approval of the study protocol must also be provided in the manuscript.

Human research participants

Policy information about studies involving human research participants

Population characteristics	Human patient serum was used freshly for osteoclastogenesis assays or processed and stored until analysis according to standard operating procedures by the MedUni Wien Biobank, a central facility included in a certified quality management system.
Recruitment	Samples were collected according to the standards of the the MedUni Wien Biobank
Ethics oversight	The MedUni Wien Biobank is approved by the local ethics committee of the Medical University Vienna (EK #559/2005).

Note that full information on the approval of the study protocol must also be provided in the manuscript.

Flow Cytometry

Plots

Confirm that:

- [x] The axis labels state the marker and fluorochrome used (e.g. CD4-FITC).
- [x] The axis scales are clearly visible. Include numbers along axes only for bottom left plot of group (a 'group' is an analysis of identical markers).
- [ ] All plots are contour plots with outliers or pseudocolor plots.
- [x] A numerical value for number of cells or percentage (with statistics) is provided.

Methodology

Sample preparation	Animals used as bone marrow donors were bred at the Medical University Vienna. Hematopoietic stem cells of the bone marrow were isolated and cultured in complete MEMα (Gibco #32561037) containing 5% Pen-Strep (Gibco #15140122) and 10% foetal calf serum (FCS, Gibco #10082147) supplemented with 100 ng/ml M-CSF (R&D Systems #416). After three days, cells were harvested, plated and cultured in Full-MEMα supplemented with 30 ng/ml M-CSF and/or 50 ng/ml RANKL (R&D Systems #462) for another 24-48 hours preharvest. Macrophage or dendritic cells were differentiated from hematopoietic stem cells cultured in presence of 30 ng/ml M-CSF (macrophages) or 20 ng/ml granulocyte macrophage colony-stimulating factor (GM-CSF, R&D #215 DCs) and 5 ng/ml IL-4 (dendritic cells), with complete medium changes on day 3, and 6. On day 7, cells were harvested. All plated cells were harvested in Accutase solution (Sigma Aldrich #A6964), resuspended in flow cytometry buffer (1xPBS with 1% FCS) and then stained for FACS analysis.<br>Spleen cells were harvested by passing through a nylon mesh and spun. Following red blood cell lysis, cells were resuspended in flow cytometry buffer and stained. Surface staining was carried out for 20 minutes at room temperature.
Instrument	CytoFLEX S Flow Cytometry (Beckman Coulter)

Software	CytExpert (Version 2.0) and FlowJo (Version 10, LLC) software
Cell population abundance	Post sample harvest, a minimum 10,000 preosteoclasts, macrophages or dendritic cells were recorded per sample. For splenocytes a minimum of 50,000 cells was recorded.
Gating strategy	Cells were gated on FSC-A/SSC-A. Where appropriate, cells were gated on live using fixable viability dye (Invitrogen #65-0865-14). Pure preosteoclast cultures derived from in vitro experiments were pre-gated on F4/80. Dendritic cells and macrophages were pregated on CD45.

☒ Tick this box to confirm that a figure exemplifying the gating strategy is provided in the Supplementary Information.
